# Prevalence and determinants of hypertensive disorders of pregnancy in Ethiopia: A systematic review and meta-analysis

**DOI:** 10.1371/journal.pone.0239048

**Published:** 2020-09-16

**Authors:** Endalamaw Tesfa, Endalkachew Nibret, Solomon Tebeje Gizaw, Yohannes Zenebe, Zewdie Mekonnen, Sefealem Assefa, Mulatu Melese, Netsanet Fentahun, Abaineh Munshea

**Affiliations:** 1 Department of Biochemistry, College of Medicine and Health Sciences, Bahir Dar University, Bahir Dar, Ethiopia; 2 Biotechnology Research Institute, Bahir Dar University, Bahir Dar, Ethiopia; 3 Department of Biology, College of Science, Bahir Dar University, Bahir Dar, Ethiopia; 4 Department of Biochemistry, College of Health Science, Addis Ababa University, Addis Ababa, Ethiopia; 5 Department of Medical Laboratory Science, College of Medicine and Health Sciences, Bahir Dar University, Bahir Dar, Ethiopia; 6 Amhara Public Health Institute (APHI), Bahir Dar, Ethiopia; 7 Department of Nutrition and Dietetics, College of Medicine and Health Sciences, Bahir Dar University, Bahir Dar, Ethiopia; University of Mississippi Medical Center, UNITED STATES

## Abstract

**Introduction:**

Hypertensive disorder of pregnancy is the second commonest causes of maternal death globally. Different public health studies were conducted on hypertensive disorder of pregnancy which presented inconsistent result. Therefore, this systematic review and meta-analysis was commenced to summarize the findings conducted in several parts of the country and to generate the nationwide representative data on the prevalence and risk factors of hypertensive disorder of pregnancy in Ethiopia.

**Methods and materials:**

Electronic databases such as PubMed, Scopus, Google Scholar, Hinari, and African Journals Online were searched for studies published in English up to March, 2020. Joanna Briggs Institute Meta-Analysis of Statistics Assessment and Review Instrument and Newcastle-Ottawa Scale were used for data extraction and quality assessment of the included studies. The meta- regression analysis was computed at 95% CI to present the pooled prevalence and risk factors of hypertensive disorder of pregnancy.

**Results:**

Thirty four studies were included in this systematic review and meta-analysis. The pooled prevalence of hypertensive disorder of pregnancy and preeclampsia in Ethiopia were 6.82% (95% CI (5.90, 7.74)) and 4.74% (95% CI (3.99, 5.49)) respectively. Maternal age ≥35 years (Adjusted Odds Ratio (AOR): 2.91 (95% CI: 1.60, 5.26)), twin pregnancy (AOR: 3.04 (95% CI: 1.89, 4.87)), previous history of preeclampsia (AOR: 5.36 (95% CI: 3.37, 8.53)), family history of hypertension (AOR: 4.01 (95% CI: 2.65, 6.07)), family history of diabetes mellitus (AOR: 3.07 (95% CI: 1.66, 7.70)), body mass index ≥25 (AOR: 3.92 (95% CI: 1.82, 8.42)), alcohol consumption (AOR: 1.77 (95% CI: 1.11, 2.83)), urinary tract infection (AOR: 4.57 (95% CI: 3.47, 6.02)), lack of nutritional counseling during antenatal period (AOR: 4.87 (95% CI: 3.36, 7.06)), lack of fruits (AOR: 3.49 (95% CI: 2.29, 5.30)), and vegetables consumption (AOR: 2.94 (95% CI: 2.01, 4.31)) were the risk factors of hypertensive disorder of pregnancy in Ethiopia.

**Conclusions:**

The pooled prevalence of hypertensive disorder of pregnancy is relatively higher compared with the previous reports. Maternal age ≥35 years, twin pregnancy, previous history of preeclampsia, family history of hypertension, family history of diabetes mellitus, body mass index ≥25, alcohol consumption, urinary tract infection, lack of fruits and vegetables during pregnancy were risk factors of hypertensive disorder of pregnancy. The governments and stakeholders should work to strengthen the antenatal care practice to include the possible risk factors of hypertensive disorders of pregnancy.

## 1. Introduction

Hypertensive disorders of pregnancy (HDP) include; preeclampsia, gestational hypertension, chronic hypertension, and chronic hypertension with superimposed preeclampsia [1]. HDP affects 5 to 10% of pregnant women worldwide and, resulted poor maternal and prenatal outcome [1, 2]. It is the second common cause of maternal death worldwide. HDP accounted about 76, 000 maternal and 500, 000 prenatal deaths globally per year [3]. According to World Health Organization (WHO) report in 2019, 295 000 maternal deaths was recorded globally due to pregnancy and child birth related causes in 2017. The risk of maternal death is 40 times higher in the least developed countries compared with European counties. Sub-Saharan African and Southern Asian countries accounted about 66% and 20% of the global maternal death, respectively [4].

Ethiopia is one of the highest estimated number of maternal deaths observed in the world that accounts about 4.8% (14, 000) of the global share in 2017 [4]. Based on the Ethiopian demographic health survey (EDHS) report in 2016, pregnancy related maternal deaths were 412 from 100, 000 live births [5]. In Ethiopia, nationwide cohort studies were not conducted that indicate the incidence and risk factors of HDP. However, different public health studies involving varied study design have been conducted on HDP and reported inconsistent results on the prevalence 1.2% [6] to 19.1% [7] and risk factors of HDP [8, 9].

Researchers identified different risk factors for HDP [9, 10]. Factors like; advanced maternal age, twin pregnancy, being primigravida, previous history of preeclampsia, family history of hypertension (HTN), family history of diabetes mellitus (DM) [11], being overweight or obesity or body mass index (BMI) ≥25 [9, 11], urinary tract infection (UTI) [12], alcohol consumption [13], lack of fruits and vegetables [14] during pregnancy increase the risk of HDP. In Ethiopia, only one systematic review and meta-analysis was published by Berhe and his colleges and reported 6.07% of HDP prevalence [15] which is lower than the prevalence of HDP in Africa which was 10% [16]. The previous review was including 17 (13 cross-sectional and 4 case-control) studies for the estimation of prevalence of HDP in Ethiopia. There is no published systematic review and Meta-analysis study that shows the risk factors of HDP in Ethiopia. Therefore, the current systematic review and meta-analysis was planned to assess the risk factors of HDP in Ethiopia. In addition, this review was planned to estimate the prevalence of HDP in Ethiopia by including more articles published since the previous review up-to March, 2020.

## 2. Methods and materials

### 2.1. Study design and search strategy

A systematic review and Meta-analysis of published and unpublished studies were conducted to assess the prevalence and risk factors of HDP in Ethiopia. We searched the following databases: Scopus, PubMed, Hinari, Google Scholar and African Journals Online (AJOL). The search was done by using the following Medical Subject Headings (MeSH) search terms; "Prevalence", "Risk Factors", "Hypertension", "Pregnancy", "Pregnancy induced hypertension", "Pre-Eclampsia" AND "Ethiopia" through using string term "AND" and "OR". A combination of MeSH terms and free terms were used for the searching strategy to conduct this systematic review and meta-analysis (S5 File). All published and unpublished studies up-to March, 2020 in Ethiopia were retrieved to be assessed for eligibility of inclusion in this review. Preferred Reporting Items for Systematic Reviews and Meta-Analyses (PRISMA) guideline was utilized to conduct this review [17].

### 2.2. Eligibility criteria

Inclusion and exclusion criteria;

1. Studies with cross-sectional, case-control and cohort designs were included.
2. Articles that report prevalence or risk factors of HDP in Ethiopia were included.
3. Published and unpublished articles written in English were included
4. Studies reporting HDP as an outcome variable were included.
5. Studies conducted in the community or in the health institution levels were included.
6. Conference papers, editorial notes and systematic reviews and meta-analyses were excluded from the study.

### 2.3. Study selection and screening

**Fig 1:**
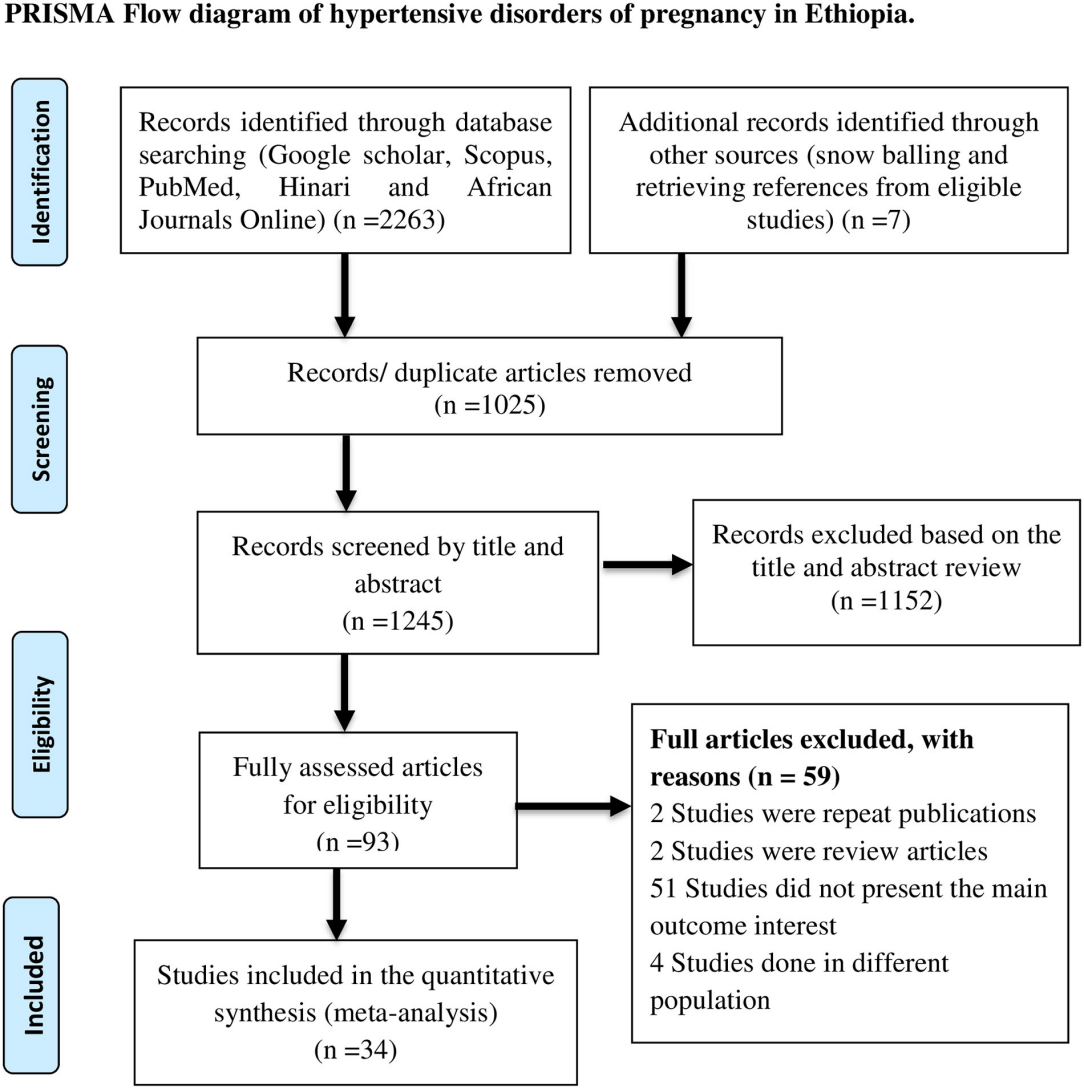
Flow diagram shows the included studies for the systematic review and meta-analysis on the prevalence and determinants of hypertensive disorders of pregnancy in Ethiopia.

All citations identified by our search strategy were exported to EndNote -X9- and duplicate articles were removed. And then the titles and abstracts of the identified articles were screened by two independent reviewers, and eligible studies were included for further review. The full texts of selected articles were retrieved and read thoroughly to ascertain the suitability prior to data extraction. In case of disagreement between the two reviewers, discussion has been held to reach consensus and the third reviewer was consulted. The search process was presented in PRISMA flow chart that clearly shows the studies that were included and excluded with sound reasons of exclusion (Fig 1) [17].

### 2.4. Definition of outcome interest

The primary outcome of this study was the prevalence of HDP. HDP includes; preeclampsia, gestational hypertension, chronic hypertension, chronic hypertension with superimposed preeclampsia and eclampsia.

- Hypertension: Systolic blood pressure ≥140mmHg and/or diastolic ≥90mmHg that is measured at least two times within four hours interval.
- Proteinuria: urinary protein excretion of ≥300mg /24-h urine sample or ≥1+ on qualitative dipstick examination or a total protein: creatinine ratio ≥30 mg/ mmol (or ≥0.3 when both are measured in mg/dL).
- Gestational hypertension: hypertension diagnosed after 20 weeks of gestation.
- Preeclampsia: gestational hypertension plus proteinuria.
- Chronic hypertension: hypertension occurs before pregnancy or diagnosed before 20 weeks of gestation for the first time.
- Chronic hypertension with superimposed preeclampsia: Occurrence of proteinuria in women with chronic hypertension before 20 weeks of gestation.
- Eclampsia: Seizures in women with hypertension that cannot be attributed to other causes [18].

The secondary outcomes of the current review were risk factors of HDP in Ethiopia (such as age, gravidity, previous history of preeclampsia, family history of hypertension and DM, BMI, nutritional counseling, alcohol drinking, UTI, eating of fruits and vegetables).

### 2.5. Quality assessment

For case-control and cohort studies, we used Newcastle-Ottawa Scale (NOS) to assess the quality of the included studies while for cross sectional studies the modified version of NOS was used to assess the quality of the studies for inclusion [19]. The NOS included 3 categorical criteria with a maximum score of 9 points. The quality of each study was rated using the following scoring algorithms: ≥7 points was considered as “Good quality”, 3 to 6 points was considered as “Fair quality”, and ≤3 point was considered as “Poor quality” (S2 File). Accordingly, in order to improve the validity of this systematic review result, we only included primary studies with fair to good quality [19].

### 2.6. Data extraction process

The data extraction was done using a tool developed by the 2014 Joanna Briggs Institute Reviewers’ Manual data extraction form [20]. The abstract and full-text were reviewed by the two independent reviewers. Data extraction includes: author’s name, publication year, study period, study design (cross-sectional, case–control and cohort), sample size, study area (region), age, gravidity, twin pregnancy, previous history of hypertensive disorders of pregnancy, family history of hypertension, family history of DM, obtaining nutritional counseling during antenatal period, BMI, consumption of alcohol, UTI, eating of fruits and vegetables and the prevalence of HDP were extracted from each article.

### 2.7. Result synthesis and statistical analysis

The data were entered into Microsoft Excel and the meta-analysis was conducted using Stata 14 software. Forest plots were used to show the prevalence and risk factors of HDP in Ethiopia at 95% CI. Subgroup analysis was done by the region (Amhara, Oromia, SNNPR, Addis Ababa, Tigray and Somali) and year of study (1991 to 2010, 2011 to 2014 and 2016 to 2020). Risk factors like; advanced maternal age, twin pregnancy, primigravidity, previous history of preeclampsia, family history of hypertension, family history of DM, BMI ≥25, alcohol consumption, UTI, eating of fruits and vegetables were assessed for their association with HDP at 95% CI.

### 2.8. Heterogeneity and publication bias

Statistical heterogeneity was estimated by using Cochrane Q, I^2^ statistic and P-value. If I^2^ statistic value < 25%, 25–50%, and ≥50% was used to declare the heterogeneity test as low, medium and high heterogeneity. In this review, random effect model was used for analysis. To cope with the reasons of heterogeneity subgroup analysis and sensitivity test were performed (S4 File) [21]. In addition, publication bias was assessed by using funnel plot asymmetry test and Newcastle-Ottawa quality assessment Scale.

## 3. Results

### 3.1. Characteristics of the included studies

A total of 2270 articles were retrieved through electronic search by using different search terms of which 1245 article were eligible for title and abstract assessment after removal of 1025 duplicate records. Out of 1245 articles screened for eligibility 1152 records were excluded by their title and abstract assessment. A total of 93 articles were undergo full- text assessment for eligibility, 59 studies were excluded due to different reasons (51 articles didn’t full fill the inclusion criteria, 4 articles were done on different population, 2 articles were repeated publications and 2 were review articles).

In this review a total of 34 studies were included. Twenty of them were cross-sectional, thirteen studies were case-control and one study was cohort. Published and unpublished studies done in Ethiopia up- to March, 2020 were included. Most regions of Ethiopia were represented in this systematic review and meta- analysis. Eleven studies were conducted in Amhara region, eight studies from Addis Ababa city, five studies from Oromia region, four studies were from Tigray region, four studies were from South Nations and Nationalities Peoples’ region (SNNPR), one study from Somali region and one was a nation based study. In the included studies the smallest sample size was 129 [22] and the maximum was 174, 561 [6]. Overall, this systematic review and meta- analysis included a total of 320,942 pregnant women in Ethiopia (Table 1).

**Table 1:** Summary of research articles included in the systematic review and meta-analysis of HDP in Ethiopia (N = 34).

No	Authors	Publication Year	Study Site (Region)	Study Design	Sample Size	Prevalence of HDP (% CI)	SE	Quality assessment
1	Hinkosa et al., 2020 [9]	2020	Oromia	Case Control	597	3.57 [2.08, 5.06]	0.759	7 points
2	Walle & Azagew, 2019 [23]	2019	Amhara	Cross -Sectional	422	16.8 [13.23, 20.37]	1.820	7 points
3	Belay and Wudad, 2019 [22]	2019	Oromia	Cross- Sectional	129	12.4 [6.71, 18.09]	2.902	7 points
4	Gudeta and Regassa, 2019 [24]	2019	SNNPR	Cross- Sectional	422	7.9 [5.33, 10.47]	1.313	5 points
5	Legesse et al., 2019 [25]	2019	Tigray	Cross- Sectional	8, 502	5.08 [4.61, 5.55]	0.238	4 points
6	Mekonnen et al., 2018 [7]	2018	Somali	Cross- Sectional	408	19.1 [15.29, 22.91]	1.946	6 points
7	Gudeta et al., 2018 [26]	2018	Oromia	Cross- Sectional	356	10.3 [7.14, 13.46]	1.611	5 points
8	Kahsay et al., 2018 [27]	2018	Tigray	Cross-Sectional	45, 329	2.98 [2.94, 3.26]	0.081	4 points
9	Wodajo and Reddy, 2016 [28]	2016	Amhara	Cross-Sectional	320	8.8 [5.70, 11.90]	1.584	7 points
10	Wagnew et al., 2016 [29]	2016	Addis Ababa	Cross-Sectional	42,963	4.2 [4.01, 4.39]	0.097	6 points
11	Shegaze et al., 2016 [30]	2016	SNNPR	Cross-Sectional	422	18.25 [14.57, 21.94]	1.880	7 points
12	Terefe et al., 2015 [31]	2015	Amhara	Cross-Sectional	8, 626	3.9 [3.49, 4.31]	0.208	5 points
13	Tessema et al., 2015 [32]	2015	Amhara	Cross-Sectional	490	8.4 [5.94, 10.86]	1.253	5 points
14	Vata et al.,2015 [33]	2015	SNNPR	Cross-Sectional	7,702	2.23 [1.90, 2.56]	0.168	5 points
15	Seyom et al., 2015 [34]	2015	Oromia	Cross-Sectional	5, 415	2.4 [1.99, 2.81]	0.208	6 points
16	Selamawit and Sisay, 2015 [35]	2015	Addis Ababa	Cross-Sectional	3,488	7.2 [6.34, 8.06]	0.438	4 points
17	Mariamawit and Shiferaw, 2014 [36]	2014	Addis Ababa	Cross- Sectional	3,351	7.8 [6.89, 8.71]	0.463	4 points
18	Wolde et al., 2011 [37]	2011	Oromia	Cross-Sectional	1, 863	8.48 [7.22, 9.75]	0.645	5 points
19	Gaym et al., 2011 [6]	2011	Nation Based	Cross -Sectional	174, 561	1.2 [1.15, 1.25]	0.026	4 points
20	Teklu and Gaym, 2006 [38]	2006	Addis Ababa	Cohort	3, 424	5.3 [4.55, 6.05]	0.383	4 points
21	Mekbebe and Ketsela, 1991 [39]	1991	Addis Ababa	Cross-Sectional	6, 766	5.14 [4.61, 5.67]	0.268	5 points
22	Hailu and Kebede, 1991 [40]	1991	Amhara	Cross-Sectional	567	12.2 [9.51, 14.89]	1.374	6 points
23	Mekie et al., 2020 [41]	2020	Amhara	Case Control	330	**---**	---	7 points
24	Fantahun and Berhane, 2019 [42]	2019	Amhara	Case Control	291	**---**	---	5 points
25	Girum et al., 2018 [43]	2018	Addis Ababa	Case Control	243	**---**	---	7 points
26	Kahsay et al., 2018 [44]	2018	Tigray	Case Control	330	**---**	---	6 points
27	Grum et al., 2017 [13]	2017	Addis Ababa	Case Control	291	**---**	---	7 points
28	Temesgen, 2017 [45]	2017	Amhara	Case Control	470	**---**	---	5 points
29	Mohammed et al., 2017 [46]	2017	Addis Ababa	Case Control	261	**---**	---	7 points
30	Aklilu et al., 2016 [47]	2016	Amhara	Case Control	831	**---**	---	4 points
31	Ayele et al., 2016 [8]	2016	SNNPR	Case Control	466	**---**	---	5 points
32	Tesfay et al., 2016 [48]	2016	Tigray	Case Control	400	**---**	---	5 points
33	Endeshaw et al., 2016 [49]	2016	Amhara	Case Control	453	**---**	---	7 points
34	Endeshaw et al., 2015 [14]	2015	Amhara	Case Control	453	**---**	---	7 points

### 3.2. Prevalence of HDP in Ethiopia

**Fig 2:**
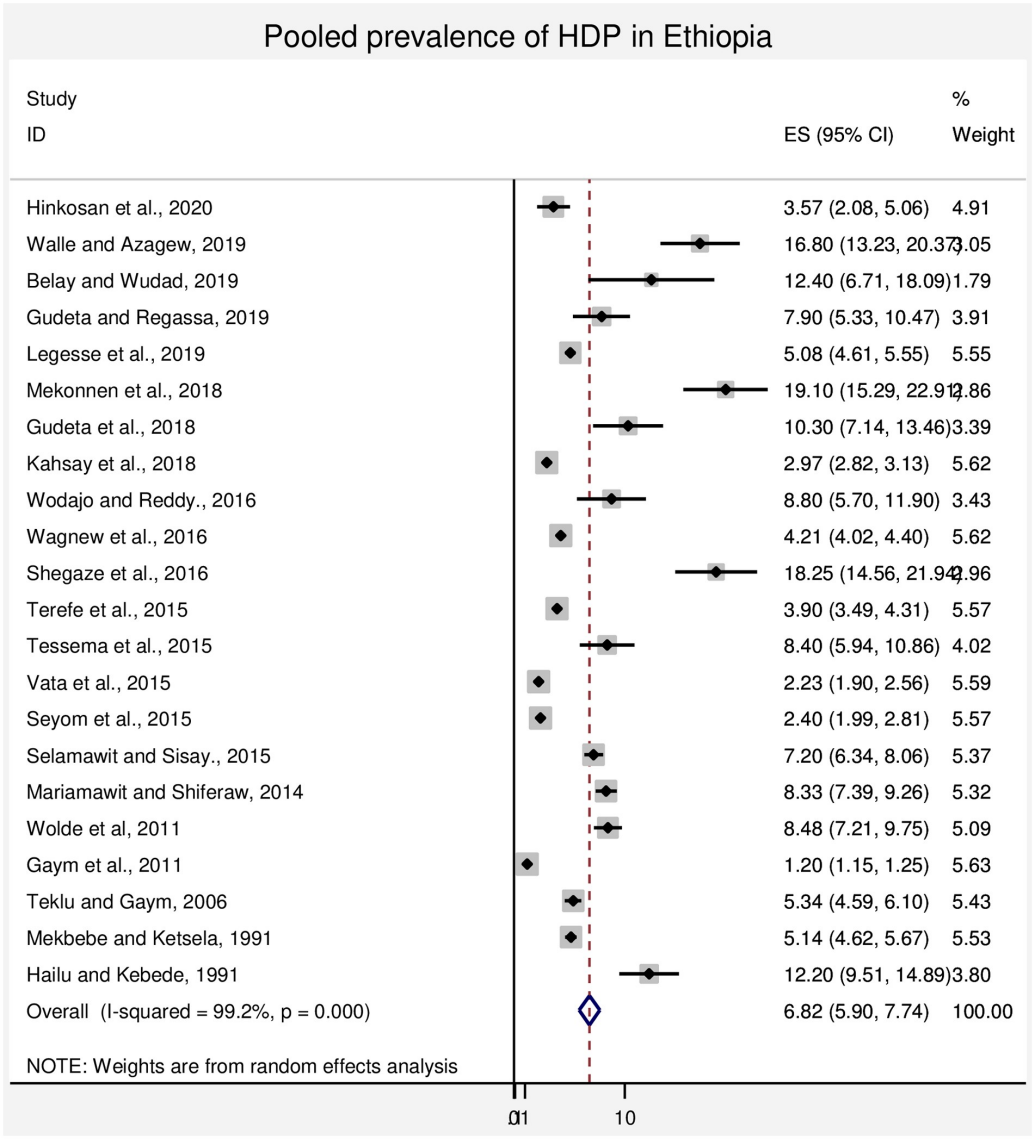
Forest plot of the pooled prevalence of HDP in Ethiopia.

A wider difference in the prevalence of HDP was observed in the studies included in this systematic review and meta-analysis. A lower prevalence (1.2%) of HDP was reported in the nation based study [6] and the higher prevalence (19.1%) of HDP was observed in the study conducted in Somali region [7] The overall pooled prevalence of HDP in Ethiopia was 6.82% (95% CI: (5.90%, 7.74%)) and summarized in Fig 2. Twenty two articles are included for the estimation of the pooled prevalence of HDP. Out of twenty two articles twenty articles were further utilized for the assessment of pooled prevalence of preeclampsia 4.74% (3.99%, 5.49%) at 95% CI.

### 3.3. Subgroup analysis of HDP prevalence in Ethiopia

The subgroup analysis of HDP prevalence by region showed the highest pooled prevalence 9.88% (5.12, 14.64)) at 95% CI in Amhara region followed by 9.29% (1.01, 17.56) at 95% CI SNNPR region. The lowest prevalence 4.02% (1.95, 6.08) at 95% CI of HDP was observed in Tigray region. The subgroup analysis of HDP by the year of study had shown the highest pooled prevalence 7.82 (6.68, 8.96) at 95% CI in the studies conducted from 2016 to 2020. This indicates that the current trend of HDP has increased compared with the studies conducted from 2006 to 2015 (Table 2).

**Table 2:** Subgroup analysis of HDP by region and year of study in Ethiopia.

Subgroup	No of studies	Pooled prevalence (95% CI)	Heterogeneity test (*I*^*2*^)	P-value
Amhara	5	9.88 (5.12, 14.64)	96.1%	<0.001
SNNPR	3	9.29 (1.01, 17.56)	97.8%	<0.001
Oromia	5	6.91 (3.51, 10.31)	96.4%	<0.001
Addis Ababa	5	6.00 (4.65, 7.35)	96.7%	<0.001
Tigray	2	4.02 (1.95, 6.08)	98.6%	<0.001
Somali	1	19.10 (15.29, 22.91)	--	--
Nation based	1	1.20 (1.15, 1.25)	--	--
**Total pooled**	**22**	**6.82 (5.90, 7.74)**	**99.2%**	**<0.001**
2016–2020	11	7.82 (6.68, 8.96)	97.3%	<0.001
2006–2015	8	5.09 (3.62, 6.57)	99.1%	<0.001
1990–2005	3	6.81 (4.89, 8.73)	92.1%	<0.001
**Total pooled**	**22**	**6.82 (5.90, 7.74)**	**99.2%**	**<0.001**

### 3.4. Risk factors of hypertensive disorders of pregnancy in Ethiopia

In this systematic review and meta- analysis different risk factors like; age, gravidity, previous history of preeclampsia, twin pregnancy, family history of hypertension, family history of DM, BMI, alcohol consumption, urinary tract infection, obtaining nutritional counseling during antenatal care (ANC) period, fruits and vegetables consumption were evaluated for their association with hypertensive disorders of pregnancy.

#### 3.4.1. Association between maternal age and HDP

**Fig 3:**
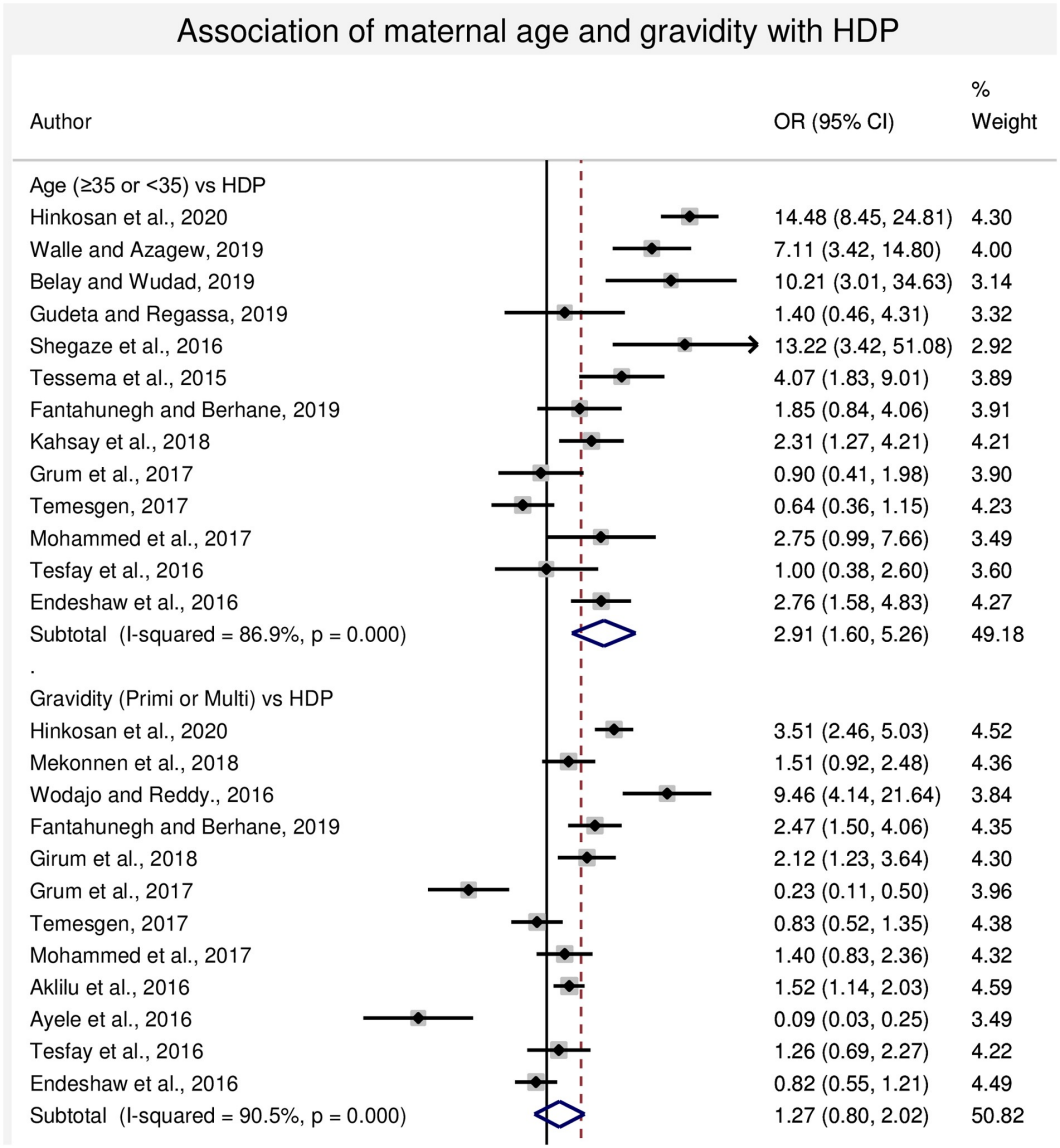
Forest plot of odds ratio for the association of maternal age and gravidity with HDP in Ethiopia.

In this sub-categorical analysis, thirteen studies were included for the assessment of age as a risk factor for HDP [9, 13, 22–24, 30, 32, 42, 44–46, 48, 50]. Seven studies had shown statistical significant association between increased maternal age with HDP [9, 22, 23, 30, 32, 44, 49]. Whereas, two studies [13, 45] had shown lower risk of HDP as maternal age increases whereas four studies [24, 42, 46, 48] reported non-significant difference between younger and older mothers. The pooled meta-regression analysis showed that there is statistical significant association in the occurrence of HDP in the maternal age ≥35 years compared with the maternal age <35 years with the odds of 2.91 (95% CI: 1.60, 5.26) (Fig 3).

#### 3.4.2. Association between gravidity and HDP

In this sub-categorical analysis, twelve studies were included for the assessment gravidity as a risk factor for HDP [7–9, 13, 28, 42, 43, 45–49]. Five of the included studies had shown statistical significant association with primigravida and HDP [9, 28, 42, 43, 47]. Whereas, four studies [8, 13, 45, 49] showed lower risk of HDP among primigravida women and three studies [7, 46, 48] showed non-significant difference. The pooled meta-regression analysis showed that there is no statistical significant difference in the occurrence of hypertensive disorders of pregnancy in the primigravida and multigravida women, OR = 1.27 (95% CI: 0.78, 2.41) (Fig 3).

#### 3.4.3. Association between twin pregnancy and HDP

**Fig 4:**
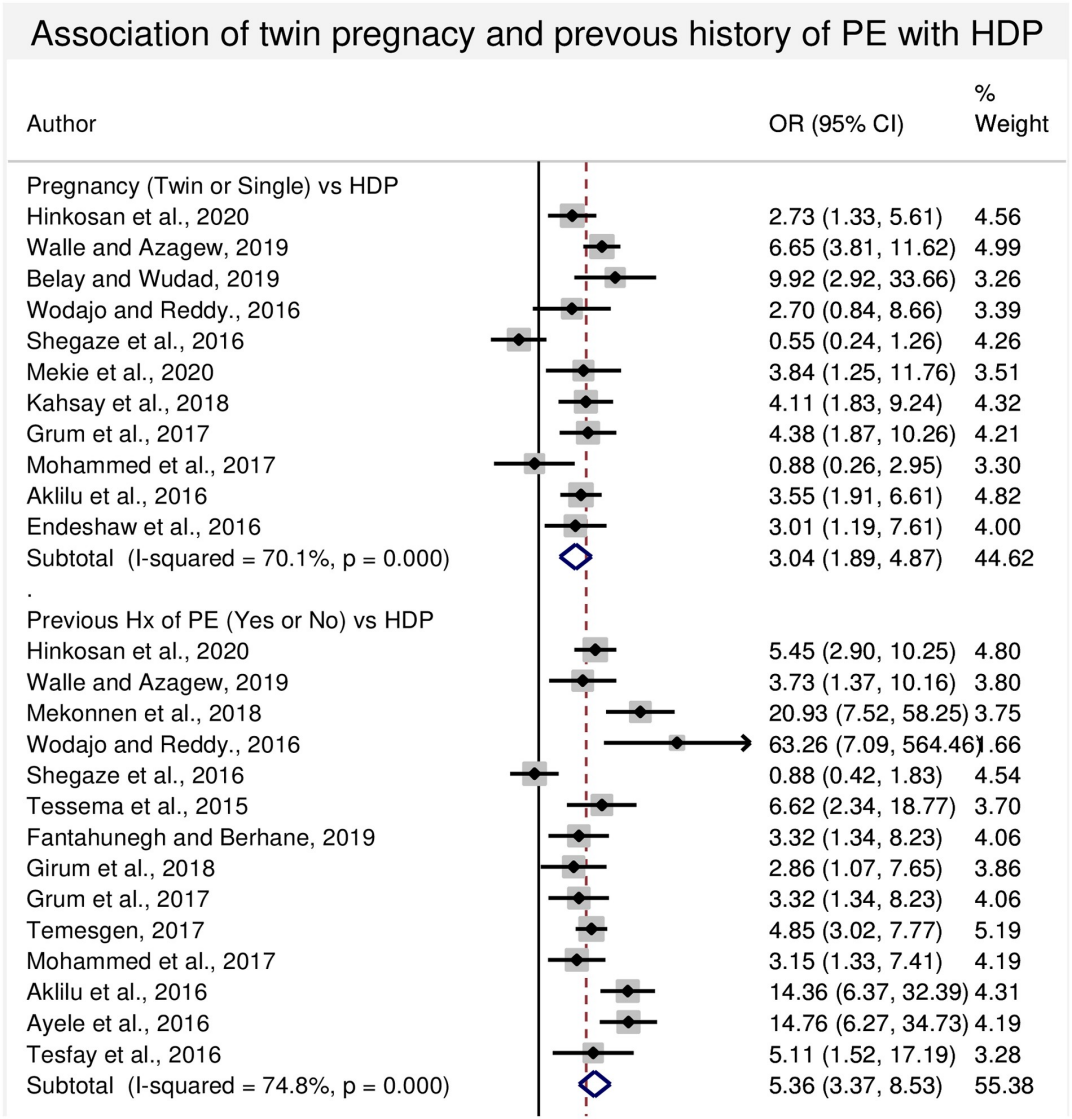
Forest plot of odds ratio for the association of twin pregnancy and previous history of preeclampsia and HDP in Ethiopia.

In this sub-categorical analysis, eleven studies were included for the assessment of twin pregnancy as a risk factor for HDP [9, 13, 22, 23, 28, 30, 41, 44, 46, 47, 49]. Eight of the included studies had shown statistical significant association among twin pregnancy and HDP [9, 13, 22, 23, 41, 44, 47, 49]. Although, two studies [30, 46] showed a lower risk of HDP among twin pregnancy, another one study [28] showed non-significant difference between the groups. The pooled meta-regression analysis showed that there is a statistical significant association between the occurrence of HDP and twin pregnancy, the odds being 3.04 (95% CI: 1.89, 4.87) times higher than that of singleton pregnancy (Fig 4).

#### 3.4.4. Association between Previous history of preeclampsia and HDP

In this sub-categorical analysis, fourteen studies were included for the assessment of previous history of preeclampsia as a risk factor for HDP [7–9, 13, 23, 28, 30, 32, 42, 43, 45–48]. Thirteen of the included studies had shown a statistical significant association between previous history of preeclampsia and HDP [7–9, 13, 23, 28, 32, 42, 43, 45–48]. However, one study showed a lower risk of HDP among previous history of preeclampsia or HDP [30]. The pooled meta-regression analysis showed that there is a statistical significant association in the occurrence of HDP and previous history of preeclampsia with the odds of 5.36 (95% CI: 3.37, 8.53) (Fig 4).

#### 3.4.5. Association between family history of hypertension and HDP

**Fig 5:**
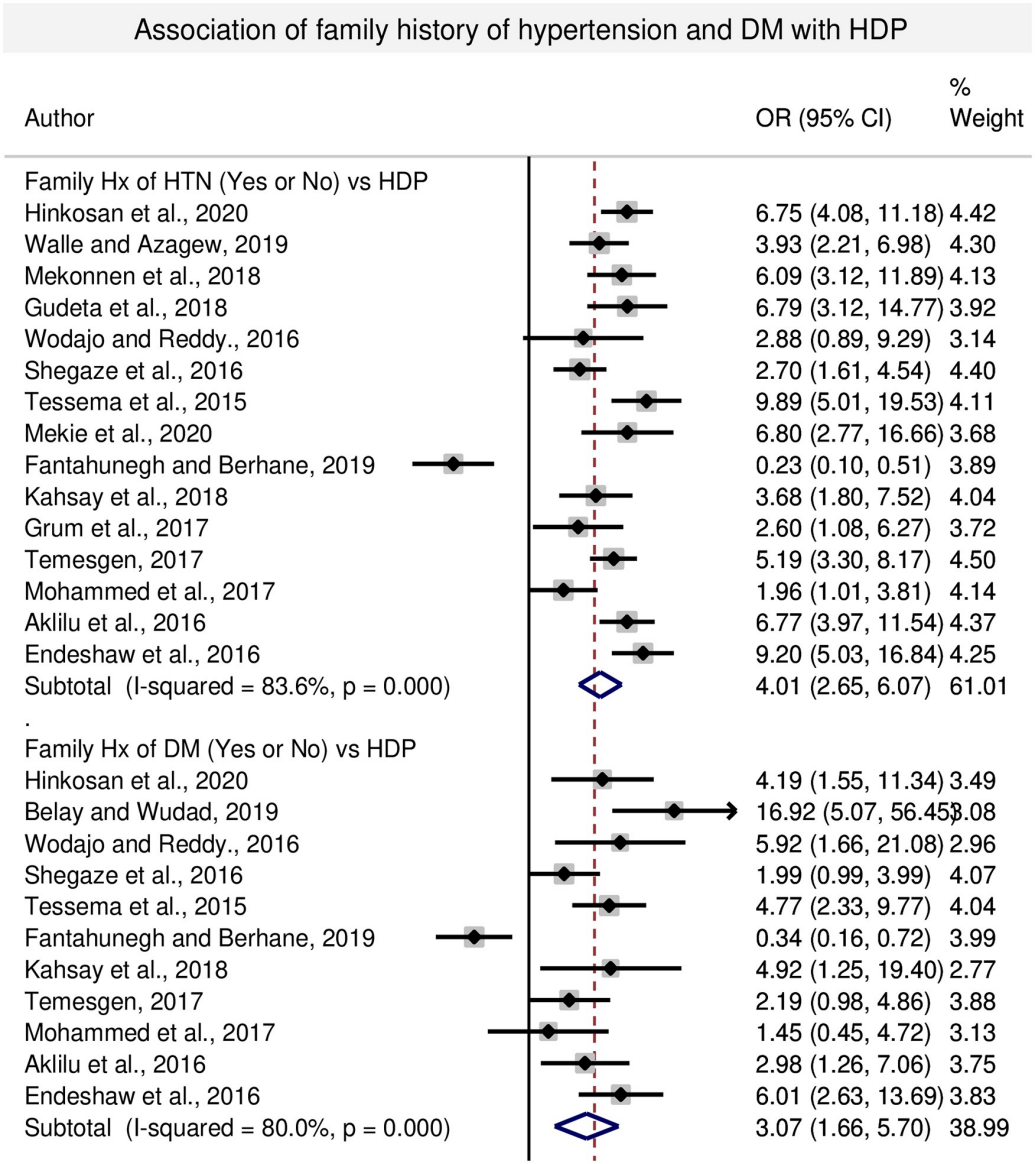
Forest plot of odds ratio for the association of family history of DM and HDP in Ethiopia.

In this sub-categorical analysis, fifteen studies were included for the assessment of family history of hypertension (HTN) as a risk factor for HDP [7, 9, 13, 23, 26, 28, 30, 32, 41, 42, 44–47, 49]. Thirteen of the included studies had shown statistical significant association of family history of HTN with HDP [7, 9, 13, 23, 26, 30, 32, 41, 44–47, 49]. Whereas, two studies [28, 42] showed lower risk and non-significant association with the women having family history of HTN and HDP. The pooled meta-regression analysis had shown statistical significant association between HDP and family history of hypertension with the odds of 4.01 (95% CI: 2.65, 6.07) compared to the women with no family history of hypertension (Fig 5).

#### 3.4.6. Association between family history of DM and HDP

In this sub-categorical analysis, eleven studies were included for the assessment of family history of diabetes mellitus as a risk factor for HDP [9, 22, 28, 30, 32, 42, 44–47, 49]. Seven of the included studies showed a statistically significant association of family history of diabetes mellitus with HDP [9, 22, 28, 32, 44, 47, 49]. While, one study [42] showed lower risk of HDP among pregnant women having family history of diabetes mellitus and three studies did not show significant association with HDP [30, 45, 46]. The pooled meta-regression analysis showed that there is a statistical significant association between the occurrence of HDP and women having family history of DM, with the odds of 3.07 (95% CI: 1.66, 5.70) (Fig 5).

#### 3.4.7. Association of BMI, nutritional counseling, UTI alcohol consumption, fruits and vegetables ingestion with HDP

**Fig 6:**
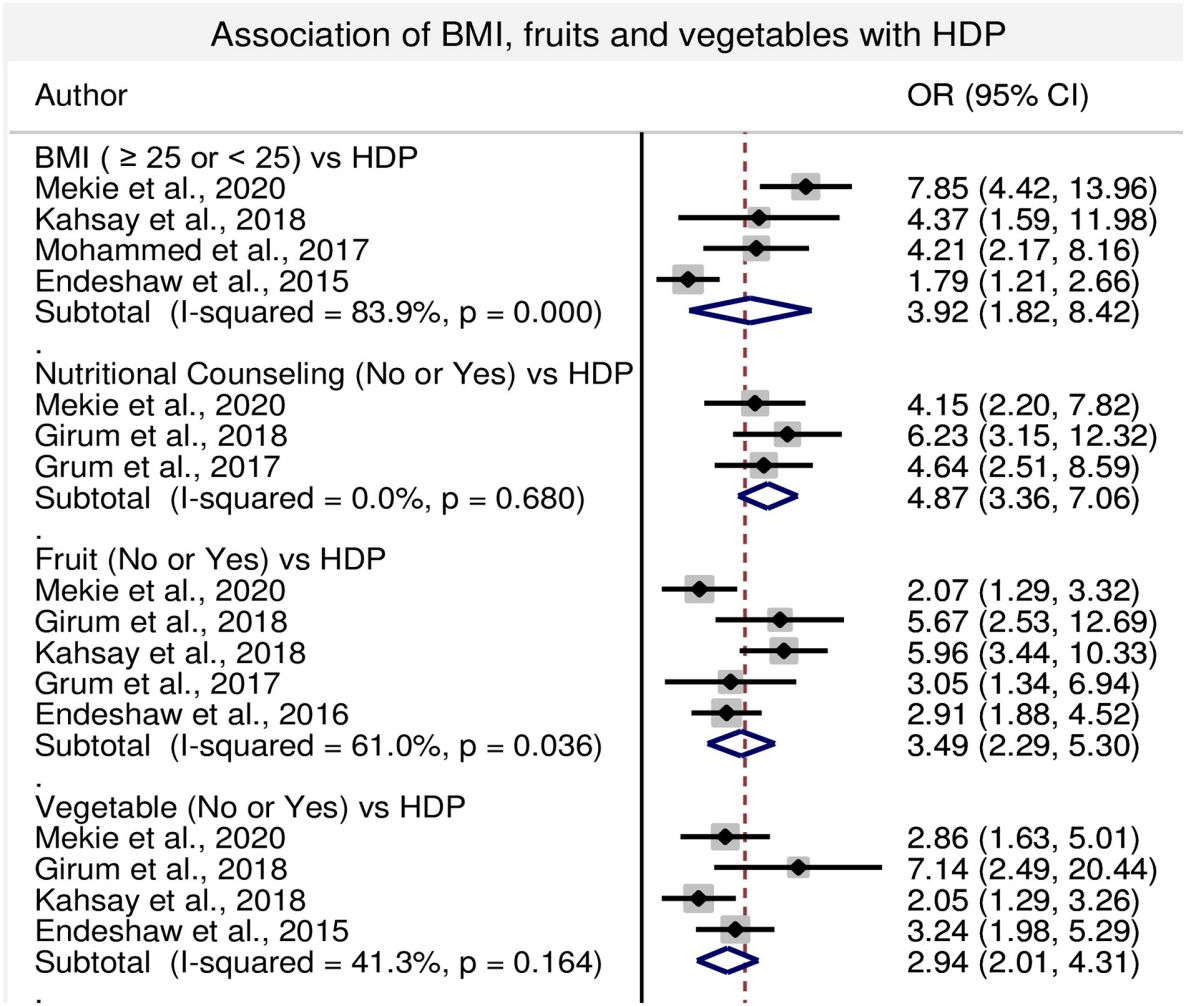
Forest plot of odds ratio for the association of BMI, nutritional counseling, fruits and vegetables consumption with HDP in Ethiopia.

**Fig 7:**
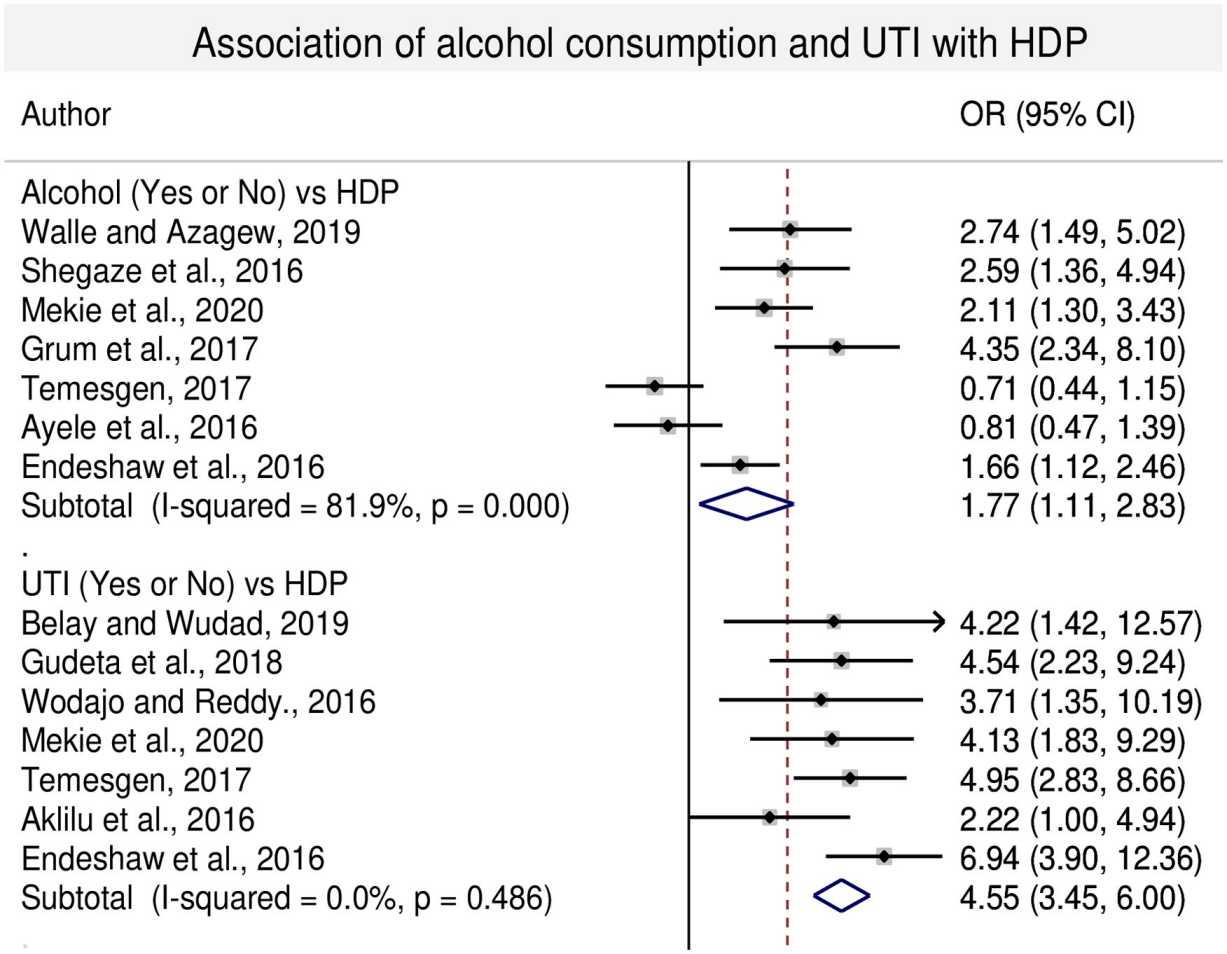
Forest plot of odds ratio for the association of alcohol consumption and UTI with HDP in Ethiopia.

In this sub-category analysis obtaining nutritional counseling during antenatal period, consumption of fruits and vegetables reduce the risk of HDP. Lack of nutritional counseling during ANC period, lack of fruits and vegetables consumption during pregnancy increase the risk of HDP with the odds of 4.87 (95% CI: 3.36, 7.06), 3.49 (95% CI: 2.29, 5.30) and 2.94 (95%: 2.01, 4.31) time, respectively. Similarly, BMI of ≥25kg/m^2^, alcohol consumption and presence of UTI during pregnancy increased the risk of HDP with the odds of 3.92 (95% CI: 1.82, 8.42), 1.77 (95% CI: 1.11, 2.83) and 4.57 (95% CI: 3.47, 6.02) times, respectively compared with the women whose BMI< 25, and women having no history of alcohol consumption and UTI during pregnancy (Figs 6 and 7).

### 3.5. Risk of publication bias

The results of this systematic review and meta-analysis were heterogeneous. To understand the cause of heterogeneity publication bias was assessed. The funnel plot figure shows the presence of possible publication bias (S3 File). This bias might be due to missing of unpublished articles in the country. The other causes might be due to the inclusion of articles with different methodology and different outcome of interest for the assessment of risk factors of HDP. Subgroup analysis and sensitivity test were done to further explore the causes of heterogeneity (S4 and S6 Files).

## 4. Discussion

This review was conducted to determine the pooled prevalence and risk factors of hypertensive disorders of pregnancy in Ethiopia. In this meta- analysis the pooled prevalence of all forms of HDP and preeclampsia were 6.82% (95% CI: (5.90%, 7.74%)) and 4.74% (95% CI (3.99, 5.49)) respectively. This is slightly higher to the previous meta- analysis report done by Berhe and his colleagues which was 6.07% (95% CI: 4.83%, 7.31%) [15]. This result found in the range of the global prevalence of HDP which is 5.2–8.2% (51). However, in this review the pooled prevalence of HDP was relatively lower than the meta-analysis done in Africa [16]. This discrepancy might be due to the number of included studies and study setting. Additionally, in this review there are retrospective cross-sectional studies that might under report the prevalence of HDP due to poor secondary data storage system [27]. In this meta-analysis the pooled prevalence of preeclampsia was 4.74% (95% CI: 3.99%, 5.49%) that is found in the range of the global report and slightly lower than the meta- analysis finding in African continent level [16, 51]. In the subgroup analysis, higher prevalence of HDP was observed in Amhara region and articles published from 2016–2020 with the prevalence of 9.88% (5.12, 14.64)) and 7.82 (6.68, 8.96), respectively (p-value <0.001) (S6 File). This difference might be due to different methodology and different outcome of interest for the assessment of risk factors of HDP in Ethiopia.

In this review, different risk factors were assessed with their association to HDP in Ethiopia. Age is an important predictor for HDP and assessed for its association with HDP by classifying maternal age ≥35 and maternal age <35 years. Maternal age of ≥35 years is almost three times more likely to develop HDP compared with the maternal age <35 years and the association were statistically significant. Similar findings were reported in the studies conducted in Kenya, Asian, China, Latin American and Caribbean women among older maternal age group with HDP compared with maternal age <35 years [11, 52–54].

The frequency of gravidity as a risk factor for HDP was assessed in this systematic review and meta-analysis. The odds of developing HDP in primigravida were 1.27 times compared with multigravida pregnant women. There was not statistical significant difference in the occurrence of HDP between primigravida and multigravida pregnant women in Ethiopia, although studies conducted in Kenya, China and Latin America had shown than women in primigravida were 2.1, 1.5 and 2.38 times more likely of developing HDP compared to multigravida pregnant women, respectively [11, 53, 54]. This difference might be attributed to the heterogeneity of the articles included in this review in respect to a particular variable. In this regard, without considering one article published by Ayele et al. (2016) in the analysis the odds of primigravidity to be associated with HDP 1.5 times compared to multigravidity and was statistically significant [8].

In this meta-analysis, twin pregnancy increased the risk of developing HDP three times more compared to singleton pregnancy and the association was statistically significant. This is similar with the studies conducted in China multiple pregnancy where they have shown a 3.68-fold higher risk of HDP compared with singleton pregnancy [55]. Similar report was observed in other multicenter trials were the odds of developing preeclampsia were 2.62 times higher compared with singleton pregnancy [56]. Multiple pregnancy causes an increased placental mass or placental hypoxia that possibly leads to the secretion of placental circulating antiangiogenic factors like;- soluble fms-like tyrosine kinase 1(sFlt1) and soluble endoglin (sEng) which antagonize the placental growth factors and vascular endothelial growth factors results in hypertension, protein and maternal syndromes [57].

Previous history of preeclampsia is an important risk factor for HDP. In this meta-analysis, women having previous history of preeclampsia were shown to develop HDP and the likelihood of its occurrence could be increased by five times as compared with HDP in those women having no previous history of preeclampsia and the association was statistically significant. Supporting evidence of our current finding was reported in the study conducted in China [11]. Similarly, family history of HTN could also increase the risk of developing HDP by four- fold compared with women having no family history of HTN. Likewise, the women having family history of DM had an increased risk of developing HDP by three-fold compared to women having no family history of DM. Thus, family history of HTN and family history of DM have shown statistical significant association with HDP. This report is consistent with the studies conducted in Swedish medical center, China and US hospitals [55, 58, 59].

Obesity is one of an important predictor for HDP or preeclampsia. In the current meta- analysis, women having BMI ≥25 had 3.9 times more risk of developing HDP compared with the women having BMI< 25 and the association was statistically significant. Supporting evidence has been found in the systematic review and meta- analysis conducted by Wang and his colleagues [60]. The exact mechanism how obesity and overweight are associated with HDP or preeclampsia is not well elucidated but obesity and overweight associated with hyperinsulinism, insulin resistance and maternal systemic inflammation. This is one of the proposed mechanisms of endothelial dysfunction, hypertension, proteinuria and multi-organ damage that occur in HDP and preeclampsia [61].

In this review, women obtained nutritional counseling during antenatal period, women consuming fruits and vegetables had lower risk of developing HDP compared with their counter- parts and the association is statistically significant. Similar report was observed in the prospective cohort study done by Timmermans and his colleagues [62]. Experimental studies in animal model proved that higher fiber and lower fat diet improve fetal development and growth through improving the antioxidant defense mechanisms that is one of the proposed pathways in the pathogenesis of HDP or preeclampsia [63]. Similar report also observed in the studies conducted in U.S.A on pregnant women in which taking higher dietary fiber was shown lower risk of preeclampsia [64].

Drinking of alcohol during the second and third trimesters of pregnancy increases the risk of preterm birth and results in different adverse consequences on the fetal development [65]. In the current review, alcohol consumption during pregnancy had shown the odds of developing HDP to be 1.77 times more compared with the women did not drink alcohol (p<0.05). The same evidence has been reported in China and Japan [55, 66]. Similarly, UTI increases the risk of developing HDP by 4.55 times more compared with those women who did not have history of UTI and the association was statistically significant. Similar results were reported by Easter et al. (2016) and Yan et al. (2018) [12, 67].

## 5. Strength and limitation

### Strength

This systematic review and meta-analysis showed the national pooled image on the risk factors of hypertensive disorders of pregnancy in Ethiopia. In addition, it produced an updated data on the prevalence of hypertensive disorders of pregnancy in Ethiopia via including more articles from the previously published reviews.

### Limitation

The search strategy may miss unpublished articles; publication bias likely high. In addition, high statistical heterogeneity was observed in this review because the inclusion of articles with different methodology and different outcome of interest for the assessment of risk factors of HDP. The included studies lack consistency to include more articles for a particular variable of risk factor that leads to small study effect. These together reduce the quality of the generated evidence.

## 6. Conclusion

The pooled prevalence of hypertensive disorders of pregnancy was found to relatively higher than what was reported previously in Ethiopia. In the subgroup analysis, the highest prevalence was observed in Amhara region and in the studies conducted between 2016 to 2020. Maternal age ≥35 years, twin pregnancy, previous history of preeclampsia, family history of hypertension, family history of diabetes mellitus, body mass index ≥25, alcohol consumption and urinary tract infection during pregnancy were significantly increased the risk of developing hypertensive disorders of pregnancy. Conversely, pregnant women obtaining nutritional counseling during antenatal period, fruit and vegetable consumption during pregnancy significantly reduce the risk of developing hypertensive disorders of pregnancy. During patient diagnosis and management clinicians will conduct detail patient evaluation to identify the risk factors of hypertensive disorders of pregnancy and to develop better treatment protocol. The governments and stakeholders should work to broaden and strengthen the antenatal care practice by involving all possible risk factors of hypertensive disorders of pregnancy in the ANC follow up guidelines. Additionally, large-scale prospective cohort studies should be needed to identify risk factors of hypertensive disorders of pregnancy in Ethiopia.

## Supporting information

S1 Checklist PRISMA check list of the review. (DOC)

S1 File Table of data extraction document. (XLSX)

S2 File Table of quality assessment. (XLSX)

S3 File Table of funnel plot. (DOCX)

S4 File Table of sensitivity test. (DOCX)

S5 File Search strategy. (DOCX)

S6 File Fig of subgroup analysis by region and year of study. (DOCX)

## Abbreviations

ANC: Antenatal care
BMI: Body mass index
CI: Confidence interval
DM: Diabetes mellitus
HDP: Hypertensive disorders of pregnancy
HTN: Hypertension
JBI-MAStARI: Joanna Briggs Institute Meta-Analysis of Statistics Assessment and Review Instrument
NOS: Newcastle-Ottawa Scale
OR: Odds ratio
SNNPR: South Nations and Nationalities Peoples’ region
U.S.A: United states of America
UTI: Urinary tract infection
