# Supplementary material for: Prevalence and determinants of hypertensive disorders of pregnancy in Ethiopia: A systematic review and meta-analysis
Source: PLoS One. 2020 Sep 16;15(9):e0239048. doi: 10.1371/journal.pone.0239048 (PMC7494091; doi:10.1371/journal.pone.0239048)
Supplement: S1 Checklist — (DOC) [file pone.0239048.s001.doc]

| **Section/topic** | **#** | **Checklist item** | **Reported on page #** |
| --- | --- | --- | --- |
| **TITLE** | | |  |
| Title | 1 | **Prevalence and determinants of hypertensive disorders of pregnancy in Ethiopia: A systematic review and Meta-analysis** | 1 |
| **ABSTRACT** | | |  |
| Structured summary | 2 | **Introduction:** hypertensive disorder of pregnancy is the second commonest causes of maternal death globally. Different public health studies were conducted on hypertensive disorder of pregnancy which presents inconsistent result. Therefore, this review was commenced to summarize the findings conducted in several parts of the country and to generate the nationwide representative data on the prevalence and determinants of hypertensive disorder of pregnancy in Ethiopia.  **Methods and Materials**: Electronic databases such as PubMed, Google Scholar, Hinari, Scopus and African Journal Online were searched for studies published in English up to March, 2020. Joanna Briggs Institute Meta-Analysis of Statistics Assessment and Review Instrument and Newcastle-Ottawa Scale were used for data extraction and quality assessment of the included studies. The meta- regression analysis was computed at 95% CI to present the pooled prevalence and determinants of hypertensive disorder of pregnancy.  **Results:** Thirty four studies were included in this review. The pooled prevalence of hypertensive disorder of pregnancy and preeclampsia in Ethiopia were 6.82% (95% CI (5.90, 7.74)) and 4.74% (95% CI (3.99, 5.49)) respectively. Age ≥35 years (Adjusted Odds Ratio (AOR): 2.91 (95% CI: 1.60, 5.26)), twin pregnancy (AOR: 3.04 (95% CI: 1.89, 4.87)), previous history of preeclampsia (AOR: 5.36 (95% CI: 3.37, 8.53)), family history of hypertension (AOR: 4.01 (95% CI: 2.65, 6.07)), family history of diabetes mellitus (AOR: 3.07 (95% CI: 1.66, 7.70)), body mass index ≥25 (AOR: 3.92 (95% CI: 1.82, 8.42)), alcohol consumption (AOR: 1.77 (95% CI: 1.11, 2.83)), urinary tract infection (AOR: 4.57 (95% CI: 3.47, 6.02)), lack of nutritional counselling during antenatal (AOR: 4.87 (95% CI: 3.36, 7.06)), not consumption of fruits (AOR: 3.49 (95% CI: 2.29, 5.30)), and vegetables (AOR: 2.94 (95% CI: 2.01, 4.31)) were determinants of hypertensive disorder of pregnancy in Ethiopia.  **Conclusions:** The pooled prevalence of hypertensive disorder of pregnancy is relatively higher. Older maternal age, twin pregnancy, previous history of preeclampsia, family history of hypertension, family history of DM, BMI≥ 25, alcohol ingestion, UTI infection, lack of fruits and vegetables during pregnancy were determinants of hypertensive disorder of pregnancy. The governments and stakeholders should work to strengthen the antenatal care practice to include the possible determinants of hypertensive disorders of pregnancy. | 2-3 |
| **INTRODUCTION** | | |  |
| Rationale | 3 | Hypertensive disorders of pregnancy (HDP) include;- preeclampsia, gestational hypertension, chronic hypertension, and chronic hypertension with superimposed preeclampsia [1]. HDP affects 5 to 10% of pregnant women worldwide and, results poor maternal and prenatal outcome [1. 2]. It is the second common cause of maternal death worldwide. HDP accounts about 76, 000 maternal deaths and 500,000 prenatal deaths globally per year [3]. According to World Health Organization (WHO) report in 2019, 295 000 maternal deaths was recorded globally due to pregnancy and child birth related causes in 2017. The risk of maternal death is 40 times higher in the least developed countries compared with European counties. Sub-Saharan African and Southern Asian countries accounted about 66% and 20% of the global maternal death, respectively [4].  Ethiopia is one of the highest estimated number of maternal deaths observed in the world that accounts about 4.8% (14, 000) of the global share in 2017 [4]. Based on the Ethiopian demographic health survey (EDHS) report in 2016, pregnancy related maternal deaths were 412 from 100, 000 live births [5]. In Ethiopia, nationwide cohort studies have not been conducted that indicate the incidence and determinants of HDP. However, different public health studies involving varied study design have been conducted on HDP and reported inconsistent results on the prevalence 1.2 % [6] to 19.1% [7] and determinants of HDP [8, 9].  Researchers have identified different risk factors for HDP [9, 10]. Factors like; advanced being old age, twin pregnancy, being primigravida, previous history of preeclampsia, family history of hypertension (HTN), family history of diabetes mellitus (DM) [11], overweight or obesity or body mass index (BMI) ≥25 [9,11], urinary tract infection (UTI) [12], alcohol ingestion [13], not consuming fruits and vegetables [14] during pregnancy increase the risk of HDP. In Ethiopia, only one systematic review and meta-analysis was published by Berhe and his colleges and reported 6.07% of HDP prevalence [15] which is lower than the prevalence of HDP in Africa which was 10% [16]. The previous review was including 17 (13 cross-sectional and 4 case-control) studies for the estimation of prevalence of HDP in Ethiopia. There is no published systematic review and Meta-analysis study that shows the determinants of HDP in Ethiopia. | 3-4 |
| Objectives | 4 | The current review was planned to assess the determinants of HDP in Ethiopia. In addition, this review was planned to estimate the prevalence of HDP in Ethiopia by including more articles published since the previous review up-to March, 2020. | 4 |
| **METHODS** | | |  |
| Protocol and registration | 5 | This protocol has been registered with the International Prospective Register of Systematic Reviews (PROSPERO). The web address and the registration number of this systematic review and meta-analysis are PROSPERO 2020 CRD42020192838 and found at https://www.crd.york.ac.uk/prospero/display_record.php?ID=CRD42020192838 | 4 |
| Eligibility criteria | 6 | Articles reporting the prevalence or determinants of HDP in Ethiopia were included. Published and unpublished studies with cross-sectional, case-control and cohort designs were included. Studies written in English reporting HDP as an outcome variable were included. Any study conducted in the community or in the health institution were included. | 5 |
| Information sources | 7 | We searched the following databases: PubMed, Hinari, Google Scholar, Scopus and African Journals Online (AJOL). All published and unpublished studies up-to March, 2020 in Ethiopia were retrieved to be assessed for eligibility of inclusion in this review. | 4 |
| Search | 8 | The search was done by using the following search terms; “Prevalence OR determinants of HDP AND Ethiopia”, “Prevalence OR determinants of pregnancy induced hypertension AND Ethiopia”, “Prevalence OR determinants of gestational hypertension AND Ethiopia”, Prevalence OR determinants of preeclampsia AND Ethiopia” separately or in combination. | 4-5 |
| Study selection | 9 | All citations identified by our search strategy were exported to EndNote -X9- and duplicate articles were removed. And then the titles and abstracts of the identified articles were screened by two independent reviewers, and eligible studies were included for further review. In case of disagreement between the two reviewers, discussion has been held to reach consensus and the third reviewer was consulted. The full texts of selected articles were retrieved and read thoroughly to ascertain the suitability prior to data extraction. The search process was presented in PRISMA flow chart that clearly shows the studies that were included and excluded with sound reasons of exclusion (Fig. 1) [17]. | 5 |
| Data collection process | 10 | Data from the selected articles were extracted by two independent reviewers by using excel data extraction sheet. Any discrepancy between the two reviewers was solved by contacting the third reviewer. | 7 |
| Data items | 11 | Data extraction includes: author’s name, publication year, study period, study design (cross-sectional, case–control and cohort), sample size, study area (region), age, gravidity, twin pregnancy, previous history of hypertensive disorders of pregnancy, family history of hypertension, family history of DM, obtaining nutritional counselling during antenatal period, BMI, consumption of alcohol, UTI, eating of fruits and vegetables and the prevalence of HDP were extracted from each article. | 7 |
| Risk of bias in individual studies | 12 | To assess risk of bias, two authors independently used the modified Newcastle Ottawa Scale for risk of bias assessment tool. Each item scored one point and discrepancies were resolved by the third reviewer. | 6 |
| Summary measures | 13 | Transformed pooled prevalence and determinants with 95% CI was the summary measure used. | 7 |
| Synthesis of results | 14 | The analysis was done through using Stata 14 statistical software. The collected data were analyzed using quantitative measures. For those variables that have significant heterogeneity random effect model analysis were done. We were computed the effect size (ES) of the prevalence and the determinants of hypertensive disorders of pregnancy in Ethiopia. In the forest plot, the box indicated weight of articles from random effect analysis. The crossed line is the 95% confidence interval (CI), the solid vertical line is zero to x-axis. | 7 |

Page 1 of 2

| **Section/topic** | **#** | **Checklist item** | **Reported on page #** |
| --- | --- | --- | --- |
| Risk of bias across studies | 15 | Sensitivity and Egger regression asymmetry test have been used for the assessment of heterogeneity and publication bias. If *I^2^* statistic value of 25%, 50%, and 75% was used to declare the heterogeneity test as low, medium and high heterogeneity. Fixed model effect was used for the analysis of non-significant heterogeneity. In case of significant heterogeneity random effect model was used for the analysis of variables [21]. | 7 |
| Additional analyses | 16 | To know the presence of heterogeneity sub-group and sensitivity analysis were performed. | 11 |
| **RESULTS** | | |  |
| Study selection | 17 | A total of 2270 articles were retrieved through electronic search by using different search terms of which 1245 article were eligible for title and abstract assessment after removal of 1025 duplicate records. Out of 1245 articles screened for eligibility 1152 records were excluded by their title and abstract assessment. A total of 93 articles were undergo full- text assessment for eligibility, 59 studies were excluded due to different reasons (51 articles didn’t full fill the inclusion criteria, 4 articles were done on different population, 2 articles were repeated publications and 2 were review articles). | 8 |
| Study characteristics | 18 | In this review a total of 34 studies were included. Twenty of them were cross-sectional, thirteen studies were case-control and one study was cohort. Published and unpublished studies done in Ethiopia up- to March, 2020 were included. Most regions of Ethiopia were represented in this systematic review and meta- analysis. Eleven studies were conducted in Amhara region, eight studies from Addis Ababa city, five studies from Oromia region, four studies were from Tigray region, four studies were from South Nations and Nationalities Peoples’ region (SNNPR), one study from Somali region and one was a nation based study. In the included studies the smallest sample size was 129 [22] and the maximum was 174, 561 [6]. Overall, this systematic review and meta- analysis included a total of 320,942 pregnant women in Ethiopia. | 8 |
| Risk of bias within studies | 19 | The risk of bias for each individual article was measured as low risk of bias, moderate risk of bias and high risk of bias and the quality assessment graded into three scales; good, fair and poor. The total score of quality assessment of each article ranges from zero to nine, which indicating that higher scores had higher quality and lower score had poor quality. In this review, we included papers that had Good and fair quality assessment result to minimize the risk of bias. | 6 |
| Results of individual studies | 20 | A wider difference in the prevalence of HDP was observed in the studies included in this systematic review and meta-analysis. A lower prevalence (1.2%) of HDP was reported in the nation based study [6] and the higher prevalence (19.1%) of HDP was observed in the study conducted in Somali region [7]. The *I^2^* test result showed high heterogeneity (*I^2^* = 99.2%, p-value = < 0.001), which is an indicative for the correct use of random effect model in our analysis. The overall pooled prevalence of HDP in Ethiopia was 6.82% (95% CI: (5.90%, 7.74%)) and summarized in **Fig. 2.** | 11 |
| Synthesis of results | 21 | In this review we had estimated the pooled prevalence of HDP at 95% CI in Ethiopia. In addition we had computed the risk factors of HDP trough estimating the odds ratio. And presented in forest plot from figure 1-9. | 9-15 |
| Risk of bias across studies | 22 | Funnel plot and sensitivity tests were used to assess the risk of bias of the included studies. Presented in the supporting files | 11 |
| Additional analysis | 23 | Determinant factor analysis was done | 12-15 |
| **DISCUSSION** | | |  |
| Summary of evidence | 24 | This review was conducted to determine the pooled prevalence and determinants of hypertensive disorders of pregnancy in Ethiopia. In this meta- analysis the pooled prevalence of all forms of HDP and preeclampsia were 6.82% (95% CI: (5.90%, 7.74%)) and 4.74% (95% CI (3.99, 5.49)) respectively. This is slightly higher to the previous meta- analysis report done by Berhe and his colleagues which was 6.07% (95% CI: 4.83%, 7.31%) [15]. This result found in the range of the global prevalence of HDP which is 5.2–8.2% [51]. However, in this review the pooled prevalence of HDP was relatively lower than the meta-analysis done in Africa [16]. This discrepancy might be due to the number of studies included in the meta- analysis and study setting. Additionally, in this review there are retrospective cross-sectional studies that might under report the prevalence of HDP due to poor secondary data storage system [27]. In this meta-analysis the pooled prevalence of preeclampsia was 4.74% (95% CI: 3.99%, 5.49%) that is found in the range of the global report and slightly lower than the meta- analysis finding in African continent level [16, 51].  In this review, different determinant factors were assessed with their association to HDP in Ethiopia. Age is an important predictor for HDP and assessed for its association with HDP by classifying the maternal age into ≥35 and <35 years. Maternal age of ≥35 years is almost three times more likely to develop HDP compared with the maternal age <35 years and the association were statistically significant. Similar findings were reported in the studies conducted in Kenya, Asian, China, Latin American and Caribbean women among older maternal age group with HDP compared with maternal age <35 years [11, 52-54].  The frequency of gravidity as a risk factor for HDP was assessed in this review. The odds of developing HDP in primigravida were 1.27 times compared with multigravida pregnant women. There was not statistical significant difference in the occurrence of HDP between primigravida and multigravida pregnant women in Ethiopia, although studies conducted in Kenya, China and Latin America had shown than women in primigravida were 2.1, 1.5 and 2.38 times more likely of developing HDP compared to multigravida pregnant women, respectively [11, 53, 54]. This difference might be attributed to the heterogeneity of the articles included in this review in respect to a particular variable. In this regard, without considering one article published by Ayele et al. (2016) in the analysis the odds of primigravidity to be associated with HDP 1.5 times compared to multigravidity and was statistically significant [8].  In this meta-analysis, twin pregnancy increased the risk of developing HDP three times more compared to singleton pregnancy and the association was statistically significant. This is jibe with the studies conducted in China multiple pregnancy where they have shown a 3.68-fold higher risk of HDP compared with singleton pregnancy [55]. Similar report was observed in other multicenter trials were the odds of developing preeclampsia were 2.62 times higher compared with singleton pregnancy (56). Multiple pregnancy causes an increased placental mass or placental hypoxia that possibly leads to the secretion of placental circulating antiangiogenic factors like;- soluble fms-like tyrosine kinase 1(sFlt1) and soluble endoglin (sEng) which antagonize the placental growth factors and vascular endothelial growth factors results in hypertension, protein and maternal syndromes [57].  Previous history of preeclampsia is an important risk factor for HDP. In this meta-analysis, women having previous history of preeclampsia were shown to develop HDP and the likelihood of its occurrence could be increased by five times as compared with HDP in those women having no previous history of preeclampsia and the association was statistically significant. Supporting evidence of our current finding was reported in the study conducted in China [11]. Similarly, family history of HTN could also increase the risk of developing HDP by four- fold compared with women having no family history of HTN. Likewise, the women having family history of DM had an increased risk of developing HDP by three-fold compared to women having no family history of DM. Thus, family history of HTN and family history of DM have shown statistical significant association with HDP. This report is consistent with the studies conducted in Swedish medical center, China and US hospitals [55, 58, 59].  Obesity is one of an important predictor for HDP or preeclampsia. In the current meta- analysis, women having BMI ≥25 had 3.9 times more risk of developing HDP compared with the women having BMI< 25 and the association was statistically significant. Supporting evidence has been found in the systematic review and meta- analysis conducted by Wang and his colleagues [60]. The exact mechanism how obesity and overweight are associated with HDP or preeclampsia is not well elucidated but obesity and overweight associated with hyperinsulinism, insulin resistance and maternal systemic inflammation. This is one of the proposed mechanisms of endothelial dysfunction, hypertension, proteinuria and multi-organ damage that occur in HDP and preeclampsia [61].  In this review, women obtained nutritional counselling during antenatal period, women consuming fruits and vegetables had lower risk of developing HDP compared with their counter- parts and the association is statistically significant. Similar report was observed in the prospective cohort study done by Timmermans and his colleagues [62]. Experimental studies in animal model proved that higher fiber and lower fat diet improve fetal development and growth through improving the antioxidant defense mechanisms that is one of the proposed pathways in the pathogenesis of HDP or preeclampsia [63]. Similar report also observed in the studies conducted in U.S.A on pregnant women in which taking higher dietary fiber was shown lower risk of preeclampsia [64].  Drinking of alcohol during the second and third trimesters of pregnancy increases the risk of preterm birth and results in different adverse consequences on the foetal development [65]. In the current review, alcohol consumption during pregnancy had shown the odds of developing HDP to be 1.77 times more compared with the women did not drink alcohol (p<0.05). The same evidence has been reported in China and Japan [55, 66]. Similarly, UTI increases the risk of developing HDP by 4.55 times more compared with those women who did not have history of UTI and the association was statistically significant. Similar results were reported by Easter et al. (2016) and Yan et al. (2018) [12, 67]. | 15-19 |
| Limitations | 25 | Strength: This systematic review and meta-analysis showed the national pooled image on the determinants of hypertensive disorders of pregnancy in Ethiopia. In addition, it produced an updated data on the prevalence of hypertensive disorders of pregnancy in Ethiopia via including more articles from the previously published reviews.  Limitation: The search strategy was limited to articles published in English, and this could lead to reporting bias. The included studies lack consistency to include all the possible determinants of hypertensive disorders of pregnancy that make us difficult to get all determinants in each study. Furthermore, presence of high statistical heterogeneity among studies conducted on the prevalence and determinants of hypertensive disorders of pregnancy was considered as limitation of this review. | 19 |
| Conclusions | 26 | The pooled prevalence of hypertensive disorders of pregnancy was found to relatively higher than what was reported previously in Ethiopia. In the subgroup analysis, the highest prevalence was observed in Amhara region and in the studies conducted between 2016 -2020. Being old age (≥35 years), twin pregnancy, previous history of preeclampsia, family history of hypertension, family history of diabetes mellitus, body mass index ≥25, alcohol consumption and urinary tract infection during pregnancy were significantly increased the risk of developing hypertensive disorders of pregnancy. Conversely, pregnant women obtaining nutritional counseling during antenatal period, fruit and vegetable consumption during pregnancy significantly reduce the risk of developing hypertensive disorders of pregnancy. During patient diagnosis and management clinicians will conduct detail patient evaluation to identify the determinants of hypertensive disorders of pregnancy and to develop better treatment protocol. The governments and stakeholders should work to broaden and strengthen the antenatal care practice by involving all possible determinants of hypertensive disorders of pregnancy in the ANC follow up guidelines. Additionally, large-scale prospective cohort studies should be needed to identify determinants of hypertensive disorders of pregnancy in Ethiopia. | 19-20 |
| **FUNDING** | | |  |
| Funding | 27 | We did not receive any fund for this study. | 20 |
