# Supplementary material for: Prevalence and determinants of hypertensive disorders of pregnancy in Ethiopia: A systematic review and meta-analysis
Source: PLoS One. 2020 Sep 16;15(9):e0239048. doi: 10.1371/journal.pone.0239048 (PMC7494091; doi:10.1371/journal.pone.0239048)
Supplement: S3 File — (DOCX) [file pone.0239048.s004.docx]

**Funnel polt shows the included studies in the meta- analysis**
