# Supplementary material for: Prevalence and determinants of hypertensive disorders of pregnancy in Ethiopia: A systematic review and meta-analysis
Source: PLoS One. 2020 Sep 16;15(9):e0239048. doi: 10.1371/journal.pone.0239048 (PMC7494091; doi:10.1371/journal.pone.0239048)
Supplement: S4 File — (DOCX) [file pone.0239048.s005.docx]

**Supporting table shows the sensitivity test of the included studies in the meta-synthsis**

| **S. No** | **Studies Omitted** | **Estimate** | **[95% Conf. Interval]** |
| --- | --- | --- | --- |
| 1 | Hinkosan et al, (2020) | 6.9917769 | 6.0465217 7.9370322 |
| 2 | Walle and Azagew, (2019) | 6.4932132 | 5.5678492 7.4185772 |
| 3 | Belay and Wudad, (2019) | 6.7178617 | 5.7904029 7.6453204 |
| 4 | Gudeta and Regassa, (2019) | 6.7738271 | 5.8370223 7.7106318 |
| 5 | Legesse et al, (2019) | 6.9003162 | 5.9662447 7.8343873 |
| 6 | Mekonnen et al, (2018) | 6.4435401 | 5.5207939 7.3662863 |
| 7 | Gudeta et al, (2018) | 6.6940365 | 5.7608972 7.6271758 |
| 8 | Kahsay et al, (2018) | 7.2787991 | 6.1781397 8.3794584 |
| 9 | Wodajo and Reddy, (2016) | 6.7477126 | 5.8130798 7.6823454 |
| 10 | Wagnew et al, (2016) | 6.9700456 | 6.0264874 7.9136038 |
| 11 | Shegaze et al, (2016) | 6.4571271 | 5.5335259 7.3807282 |
| 12 | Terefe et al, (2015) | 7.0049462 | 6.0513711 7.9585218 |
| 13 | Tessema et al, (2015) | 6.750062 | 5.8136063 7.6865177 |
| 14 | Vata et al, (2015)\| | 7.1510735 | 6.1690316 8.1331158 |
| 15 | Seyom et al, (2015)\| | 7.1190157 | 6.1500587 8.0879726 |
| 16 | Selamawit and Sisay, (2015) | 6.7665114 | 5.8384962 7.6945262 |
| 17 | Mariamawit and Shiferaw, (2014) | 6.6914659 | 5.7698507 7.6130815 |
| 18 | Wolde et al, (2011) | 6.7075911 | 5.7764339 7.6387486 |
| 19 | Gaym et al, (2011)\| | 6.8455987 | 6.0542436 7.6369538 |
| 20 | Teklu and Gaym, (2006) | 6.8948956 | 5.9547296 7.8350616 |
| 21 | Mekbebe and Ketsela, (1991)\| | 6.9001942 | 5.9640684 7.8363199 |
| 22 | Hailu and Kebede, (1991) | 6.5963273 | 5.6655335 7.5271206 |
|  | Combined | 6.8222515 | 5.9013923 7.7431108 |
