# Supplementary material for: Prevalence and determinants of hypertensive disorders of pregnancy in Ethiopia: A systematic review and meta-analysis
Source: PLoS One. 2020 Sep 16;15(9):e0239048. doi: 10.1371/journal.pone.0239048 (PMC7494091; doi:10.1371/journal.pone.0239048)
Supplement: S5 File — (DOCX) [file pone.0239048.s006.docx]

**This is an example of the searching strategy for the PubMed data base**

1. (((("Prevalence"[Mesh]) OR "Risk Factors"[Mesh]) AND "Hypertension"[Mesh]) AND "Pregnancy"[Mesh]) AND "Ethiopia"[Mesh]
2. (((("Prevalence"[Mesh]) OR "Risk Factors"[Mesh]) AND "Pregnancy"[Mesh]) AND "Hypertension"[Mesh]) AND "Ethiopia"[Mesh]
3. ((("Prevalence"[Mesh]) OR "Risk Factors"[Mesh]) AND "Pre-Eclampsia"[Mesh]) AND "Ethiopia"[Mesh]
4. ((("Prevalence"[Mesh]) OR "Risk Factors"[Mesh]) AND "Hypertension, Pregnancy induced"[Mesh]) AND "Ethiopia"[Mesh]
5. ((("Prevalence"[Mesh]) OR "Risk Factors"[Mesh]) AND "Pre-Eclampsia"[Mesh]) AND "Ethiopia"[Mesh]

In addition we used a combination of these MeSH terms and free terms for the searching strategy to conduct this systematic review and meta-analysis.
